# Dystrophic calcinosis cutis associated with systemic lupus erythematosus: a case report

**DOI:** 10.11604/pamj.2021.40.258.28215

**Published:** 2021-12-22

**Authors:** Filipa Costa Sousa, Mariana Figueiras, Ana Rita Parente, Sónia Santos, Mafalda Miranda, Mónica Teixeira, Teresa Mendonça

**Affiliations:** 1 Department of Clinical Immunology, Centro Hospitalar Universitário do Porto, Porto, Portugal,; 2 Department of Internal Medicine, Centro Hospitalar Tondela Viseu, Viseu, Portugal,; 3 Department of Internal Medicine, Centro Hospitalar Entre Douro e Vouga, Santa Maria da Feira, Portugal

**Keywords:** Dystrophic calcinosis cutis, systemic lupus erythematosus, calcium channel blockers, case report

## Abstract

Calcinosis cutis is a rare and potentially disabling condition characterized by calcium deposition in soft tissues. When associated with autoimmune connective tissue diseases, calcinosis cutis is classified as Dystrophic Calcinosis Cutis (DCC), being its occurrence in systemic lupus erythematosus (SLE) patients fairly uncommon. We report a case of DCC in a 49 years old woman with eleven years evolution SLE that presented with a two years history of multiple painful skin lesions, some of them ulcerated and exhibiting a chalky white-yellow floor, in both hands, forearms, thighs, buttocks, abdomen and left breast. The pelvic X-ray showed soft tissue calcifications and the skin biopsy confirmed the diagnosis of DCC. The patient was treated with diltiazem 240mg/day and a significant regression of the lesions and associated pain was observed. Dystrophic calcinosis cutis is often a painful and disrupting condition in which timely diagnosis and treatment may be quite challenging.

## Introduction

Calcinosis cutis is a rare, chronic and potentially disabling condition characterized by calcium deposition in soft tissues like skin, subcutaneous tissue, joints, ligaments and muscles [1,2]. Depending on the underlying etiology, there are four subtypes of calcinosis cutis: dystrophic, metastatic, iatrogenic and idiopathic [1,3]. When associated with autoimmune connective tissue diseases, calcinosis cutis is classified as DCC. Systemic sclerosis and dermatomyositis are the most frequent conditions associated with DCC, being its association with SLE fairly uncommon [1,3,4]. Due to its rarity, diagnosis and treatment of this condition may be quite challenging. In this report, we present a case of the rare occurrence of DCC in a patient with SLE, highlighting the clinical presentation, diagnostic workup and treatment.

## Patient and observation

**Patient information:** a 49-year-old woman presented at the outpatient clinic with a two years history of several painful lesions in both hands, forearms, thighs, buttocks, abdomen and left breast. The patient had 11 years evolution SLE with cutaneous, articular and hematologic manifestations: three years evolution lupus panniculitis, malar rash, Raynaud phenomenon, polyarthritis and lymphopenia. She was taking hydroxychloroquine 400mg/day; subcutaneous methotrexate 20mg/week; prednisolone 10mg/ day; nifedipine 60mg/day; folic acid 5mg/day; onagra oil and vitamin C.

**Figure 1:**
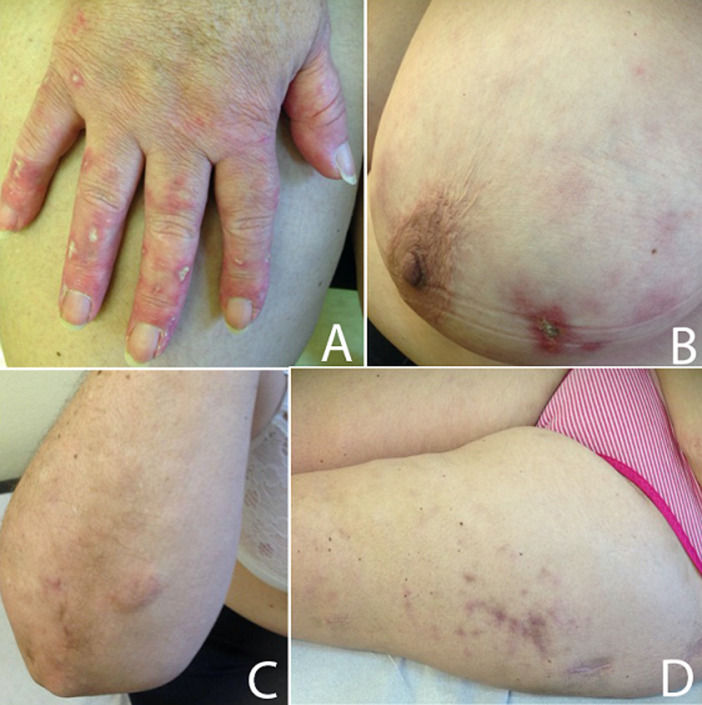
calcinosis cutis lesions at presentation; A) cutaneous ulcerated lesions with chalky white-yellow floor and erythematous edge in the dorsum of the right hand; B) left breast; C) subcutaneous nodules in the right forearm; D) erythematous papules in left thigh

**Clinical findings:** on physical examination, the patient presented with cutaneous ulcerated lesions with chalky white-yellow floor and erythematous edge in the dorsum of the right hand and left breast, subcutaneous nodules, of 2cm of larger diameter, in the right forearm and erythematous papules in left thigh (Figure 1). All lesions were painful at palpation.

**Timeline of current episode:** October 2018: the patient is seen in the outpatient clinic and lesions are assessed; December 2018: analytical and imagiologic study is conducted. February 2019: skin biopsy. April 2019: switch from nifedipine to diltiazem 240mg/day; October 2019: lesions regression is observed.

**Figure 2:**
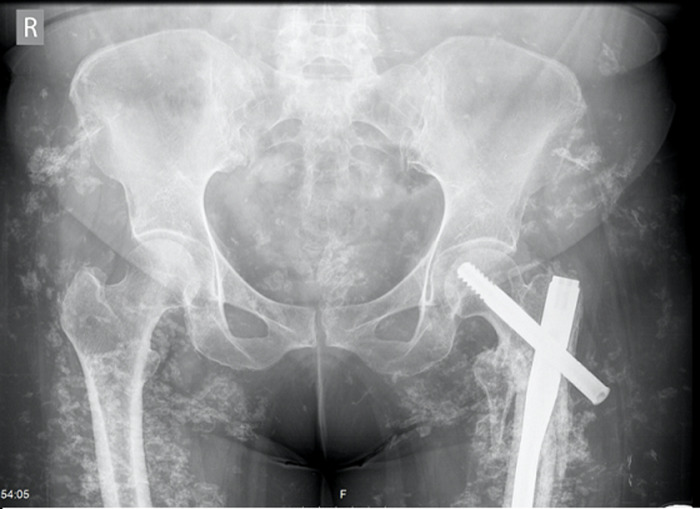
pelvic X-ray showing extensive soft tissue calcification

**Diagnostic assessment:** conditions possibly associated with calcinosis cutis, other than SLE, such as kidney injury, phosphocalcic metabolism disorders, sarcoidosis and neoplasms were ruled out. The pelvic X-ray showed extensive soft tissue calcification (Figure 2) and skin biopsy revealed dermis calcium deposits.

**Diagnosis:** based on consistent clinical findings and diagnostic workup, the diagnosis of DCC was established.

**Therapeutic interventions:** Nifedipine was suspended and the patient was treated with diltiazem 240mg/day.

**Figure 3:**
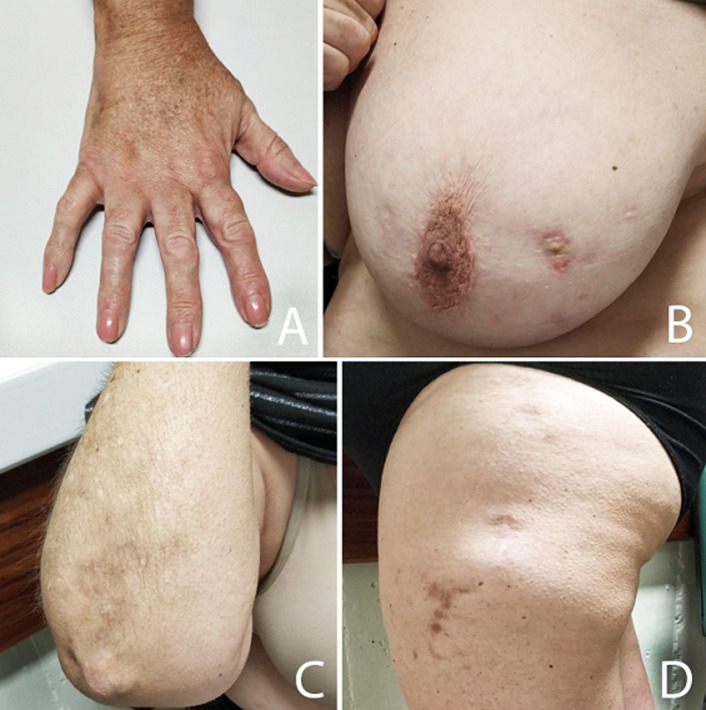
calcinosis cutis lesions after six months of treatment with diltiazem; A-D) resolution of inflammatory process and skin scaring is observed in all locations previously affected by DCC

**Follow-up and outcome of interventions:** significant regression of the lesions and associated pain was observed at six months follow-up (Figure 3).

**Patient perspective:** “since I've been taking this new medication, I've noticed that my skin is better. These wounds are healing and don't hurt anymore”.

**Informed consent:** the patient has given informed consent to the publishing of this case report. She has also, kindly and willingly, collaborated in the photographic documentation of her lesions, being aware of the publishing purposes.

## Discussion

In spite of being most frequently seen in association with autoimmune connective tissue diseases such as systemic sclerosis and dermatomyositis, dystrophic calcinosis cutis can also be associated with neoplasms, collagen/elastin disorders, among other conditions [1,3]. Dystrophic calcinosis cutis associated with SLE is fairly uncommon and usually develops after long-standing systemic disease [1,3,4]. Balin *et al*. reported a mean time to DCC diagnosis of 21.5 years after the diagnosis of SLE but a much shorter mean time in patients with lupus panniculitis (4,8 years) [3]. In the present clinical case, the time to DCC diagnosis was shorter than the reported mean: 11 years after SLE and 3 years after lupus panniculitis diagnosis. Pathophysiology of calcinosis cutis is not yet fully understood. Whereas phosphocalcic disturbances play an important role in the metastatic and iatrogenic subtypes of calcinosis cutis, normal serum calcium and phosphate levels are observed in idiopathic and dystrophic subtypes [1,4,5]. Abnormal tissue structure as a result of chronic tissue damage or defective collagen/ elastin synthesis, reduced vascularization and hypoxia seem to have a contribute on DCC pathophysiology [1-4]. The extent of calcification is highly variable, ranging from limited areas of calcification, often asymptomatic, to widespread involvement, usually leading to great disability [1,2,4]. These lesions can ulcerate and discharge white, chalky materials with subsequent inflammation and pain. Suspicion of calcinosis cutis should arise in the presence of these kinds of lesions. Complications like infection, tissues atrophy, joint constructures, deformities, issues of aesthetic and functional impotence may occur secondarily [2,3,5].

As a disruptive condition that may cause significant morbidity, DCC requires a timely diagnosis [1]. However, due to its rarity, the diagnosis of DCC may be quite challenging. This difficulty is well exemplified by the two years timespan between symptoms onset and the definitive diagnosis of DCC in the present clinical case. Possible underlying causes of calcinosis cutis, such as phosphocalcic disturbances, iatrogenesis, kidney injury, malignances or autoimmune connective tissue diseases must be accessed. In this regard, a thorough history taking is crucial and, depending on the patient´s circumstance, analysis including serum calcium and phosphorus levels, creatinine, parathyroid hormone, vitamin D, angiotensin-converting enzyme, and serum protein electrophoresis may be helpful [1]. The X-ray is a useful tool to support the diagnosis and to determine the extent of tissues calcifications [1]. Skin biopsy of an involved area is particularly valuable in the case of diagnosis uncertainty, as it confirms the diagnosis [3]. Although multiple medical and procedural techniques have been investigated, they lack clear evidence of effectiveness [1,4]. Therefore, there is no gold standard therapy available for DCC [1,4]. Nevertheless, many procedural and medical therapies, often used in combination, may have a place. Treatment strategy should be individualized, aiming the optimization of the underlying condition, if any, symptoms relief and functional recovery [1,4]. Some of the procedural options that may be applied in localized lesions are surgical excision, laser therapy and extracorporeal shock wave lithotripsy [1]. Medical therapies such as diltiazem, minocycline, and colchicine have also shown benefit in the treatment of calcinosis cutis patients [1,3,4].

Calcium channel blockers (CCB) have been the most widely used medical treatment for calcinosis cutis. Balin *et al*. reported CCB as the most efficacious medical therapy on a retrospective study that included 78 patients with DCC [3]. Although the mechanism by which this class of drugs acts in calcinosis cutis is unclear, it is hypothesized that CCB decrease cellular calcium influx and, thus, reduce calcium crystallization in the affected tissues [1-4]. Calcium channel blockers may also bring benefit by relieving vascular insufficiency in the tissue surrounding the lesions, thereby lessening the burden of tissue damage and calcium deposition [1]. Diltiazem is the most studied CCB in the treatment of calcinosis cutis and have been suggested as first line therapy in the dose of 240-480 mg/day by some authors [1,3,4]. The use of others CCB is less stablished in literature. Based on a partial response to amlodipine in one patient, reported by Balin *et al*., Boulman *et al*. suggest the use of this drug as an alternative in patients with cardiologic contraindications for diltiazem [2,3]. There are, to the best of our knowledge, no data regarding the use of nifedipine in the treatment of calcinosis cutis. Also, our patient developed this condition under the use of this drug. These facts combined with the scientific evidence on the use of diltiazem, have substantiated the decision to switch from nifedipine to diltiazem. Beyond presenting a case of the quite rare association between DCC and SLE, the present report also brings us a detailed photographic documentation of the skin lesions as well as X-ray typical findings. With this case report we hope to contribute to an easier recognition and diagnosis of this disease in the clinical practice. Furthermore, we report a case of a therapeutic success in an area of sparce scientific evidence.

## Conclusion

Dystrophic calcinosis cutis associated with SLE is a rare and potentially disabling condition whose diagnosis and treatment may be quite challenging. Clinicians´ awareness of DCC manifestations and an adequate diagnostic work-up may be the key to overcome this issue. Despite lacking clear evidence of effectiveness, some treatment options may have great impact in symptoms management and, thus, positively influence the quality of life of our patients, as the present case report demonstrates.
